# Hierarchical Regulatory Networks Reveal Conserved Drivers of Plant Drought Response at the Cell‐Type Level

**DOI:** 10.1002/advs.202415106

**Published:** 2025-03-16

**Authors:** Moyang Liu, Yuanyuan Xu, Yue Song, Dongying Fan, Junpeng Li, Zhen Zhang, Lujia Wang, Juan He, Cheng Chen, Chao Ma

**Affiliations:** ^1^ School of Agriculture and Biology Shanghai Jiao Tong University Shanghai 200240 China

**Keywords:** AlphaFold 3, CIPK‐CBL, conserved mechanism, drought response, key driver, multi omics

## Abstract

Drought is a critical environmental challenge affecting plant growth and productivity. Understanding the regulatory networks governing drought response at the cellular level remains an open question. Here, a comprehensive multi‐omics integration framework that combines transcriptomic, proteomic, epigenetic, and network‐based analyses to delineate cell‐type‐specific regulatory networks involved in plant drought response is presented. By analyzing nearly 30 000 multi‐omics data samples across species, unique insights are revealed into conserved drought responses and cell‐type‐specific regulatory dynamics, leveraging novel integrative analytical workflows. Notably, *CIPK23* emerges as a conserved protein kinase mediating drought tolerance through interactions with *CBL4*, as validated by yeast two‐hybrid and BiFC assays. Experimental validation in *Arabidopsis thaliana* and *Vitis vinifera* confirms the functional conservation of *CIPK23*, which enhances drought resistance in overexpression lines. In addition, the authors’ causal network analysis pinpoints critical regulatory drivers such as *NLP7* and *CIPK23*, providing insights into the molecular mechanisms of drought adaptation. These findings advance understanding of plant drought tolerance and offer potential targets for improving crop resilience across diverse species.

## Graphical abstract

This study presents a comprehensive multi‐omics framework to uncover cell‐type‐specific regulatory networks in plant drought responses. By integrating transcriptomic, proteomic, and epigenetic data from nearly 30 000 samples, key regulators such as CIPK23 and NLP7 are identified, revealing insights into conserved drought tolerance mechanisms and offering potential targets for enhancing crop resilience.

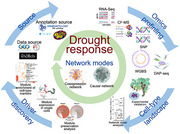

## 1 Introduction

Drought is among the most pressing environmental challenges threatening plant growth and crop productivity worldwide.^[^ ^1^ ^]^ As global climate patterns shift, water availability becomes increasingly unpredictable, exacerbating the need for crops that can thrive under water‐limited conditions.^[^ ^2^ ^]^ Plants have evolved intricate regulatory mechanisms to perceive and respond to drought stress, which help them maintain cellular homeostasis and ensure survival in arid environments.^[^ ^3^ ^]^ These responses span various biological levels, from morphological adaptations, such as enhanced root elongation, to molecular modifications, including changes in gene expression, protein function, and metabolite accumulation.^[^ ^4^ ^]^ Understanding these complex, multi‐layered drought responses is essential for developing crops that can withstand drought conditions, a goal of paramount importance in light of current and future climate changes.

Over the last decade, the rapid advancement of multi‐omics technologies—genomics, transcriptomics, proteomics, and epigenomics—has significantly deepened our understanding of how plants respond to drought.^[^ ^1^, ^5^ ^]^ These high‐throughput techniques allow for the comprehensive analysis of gene expression patterns, protein interactions, and epigenetic modifications, thereby offering insights into the molecular networks underlying stress responses.^[^ ^6^ ^]^ Despite these advances, most drought‐related research focuses on bulk tissue samples, which tends to obscure the nuanced, cell‐type‐specific regulatory dynamics that are essential for a holistic understanding of how plants adapt to drought. Different cell types within a plant may respond to drought in unique ways, contributing variably to the plant's overall drought tolerance. However, much remains to be discovered about the cell‐specific regulatory mechanisms that govern these differential responses.^[^ ^7^ ^]^ A clearer understanding of these processes at the cellular level will be critical for enhancing crop resilience.

Significant strides have been made in identifying the genes, proteins, and signaling pathways that confer drought tolerance in plants.^[^ ^4^, ^8^ ^]^ Many of these discoveries have been facilitated by genome‐wide association studies (GWAS), RNA sequencing (RNA‐seq), and proteomic analyses. These studies have pinpointed drought‐responsive genes, such as those encoding transcription factors, protein kinases, and other regulatory proteins, as well as downstream metabolic pathways that mediate drought tolerance.^[^ ^9^ ^]^ However, despite the volume of data generated, there is a persistent gap in understanding how these elements interact within specific cell types and across multiple omic layers. Existing studies often lack the resolution needed to distinguish between responses occurring in different cell types, and they rarely integrate findings from different omic levels to form a cohesive picture of drought response mechanisms. For example, transcriptomic data may reveal genes that are differentially expressed under drought conditions, but without corresponding epigenomic or proteomic data, it is challenging to determine how these genes are regulated or how they affect downstream protein activity. Another limitation of previous drought research is their frequent reliance on model organisms such as *Arabidopsis thaliana*, which, while useful, limit the generalizability of findings to other crop species. Drought responses are highly species‐specific, with different crops exhibiting distinct adaptive mechanisms.^[^ ^10^ ^]^ Thus, an integrative, cross‐species approach is required to uncover both conserved and species‐specific regulatory mechanisms. By employing multi‐omics integration, we can begin to map the complex networks that govern drought responses across various plant species, providing a foundation for improving drought tolerance in a wide range of crops.

This study aims to unravel the complex regulatory networks of plant drought response by integrating multi‐omics data at unprecedented cell‐type resolution. Using multi‐omics data integration, including advancements such as AlphaFold for protein interaction predictions, we uniquely map the conserved and hierarchical regulatory mechanisms driving drought tolerance. Notably, we demonstrate the pivotal role of the CIPK gene in coordinating a conserved response to drought across different species. Collectively, our findings underscore the conservation of a common drought response network. This knowledge not only enriches our understanding of the molecular basis of drought tolerance but also paves the way for the development of targeted strategies for crop improvement. As global water scarcity becomes an increasingly urgent issue, the ability to enhance crop drought tolerance through a multi‐omics approach represents a critical advancement in sustainable agriculture.

## 2 Results

### 2.1 A Pipeline of Multiscale Gene Network Analysis of Drought Response

**Figure 1:**
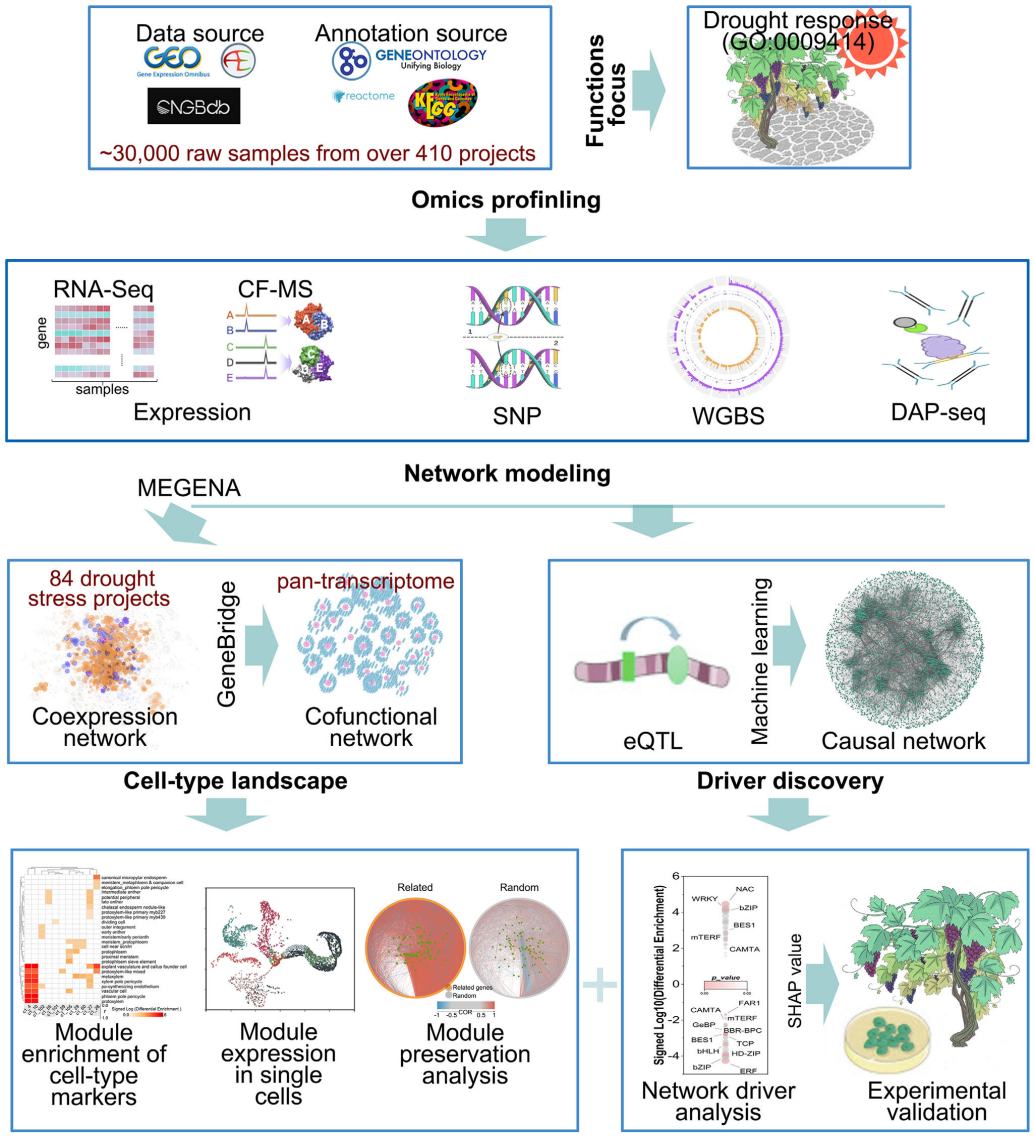
Multi‐stage workflow for identifying conserved and species‐specific drivers of plant drought response. This schematic outlines the multi‐stage approach used to uncover molecular mechanisms of drought response in plants. The analysis integrated nearly 30 000 raw data samples from over 410 projects, covering various omics data such as transcription levels, regulation, methylation, and protein interactions. The MEGENA framework was applied to 84 drought stress datasets to construct co‐expression networks and define the cell‐type landscape. Drought‐responsive genes were identified using the G‐MAD tool; while, co‐functional networks were inferred with the M‐MAD tool. Network conservation was validated using *Arabidopsis* data from the 1001 Genome Project, and cell‐type‐specific functions were revealed through enrichment of marker genes in single‐cell RNA‐seq data. Further, species‐specific and conserved functions were explored through the OneKP project. The integration of genotype and gene expression data enabled eQTL enrichment and causal network analysis, leading to the identification of causal associations and regulatory driver genes. Experimental validation confirmed the roles of these network drivers in drought response. See also Table S1, Supporting Information.

Our analysis included nearly 30 000 raw data samples collected from over 410 projects encompassing diverse targets and methodologies, including transcription levels, transcriptional regulation, methylation patterns, and protein interactions (**Figure** **1**; Table S1, Supporting Information). Our workflows followed a multi‐stage approach (Figure 1): First, we applied MEGENA^[^ ^11^ ^]^ to 84 drought stress projects (the remaining projects were used for other subsequent analyses) to map the network structure and delineate the cell type landscape. To identify drought‐response genes, we utilized the G‐MAD tool from the GeneBridge toolkit;^[^ ^12^ ^]^ while, the M‐MAD tool was employed to infer co‐functional networks involved in the drought response. For network conservation validation, we primarily analyzed the *Arabidopsis* cohort from the 1001 Genome Project (PRJNA273563).^[^ ^13^ ^]^ We identified cell type‐specific functions through enrichment analysis of cell type marker genes in the scRNA‐seq dataset.^[^ ^14^ ^]^ A network conservation analysis using the OneKP project^[^ ^15^ ^]^ revealed both conserved and species‐specific functions. Following this, we integrated genotype and gene expression data to perform eQTL enrichment and causal network analysis,^[^ ^16^ ^]^ allowing us to detect causal associations and pinpoint driver genes within the drought response synergistic network. Finally, we conducted experimental validation to confirm the regulatory roles of these identified network driver genes.

### 2.2 Cell‐Type‐Specific Drought Response Functions

**Figure 2:**
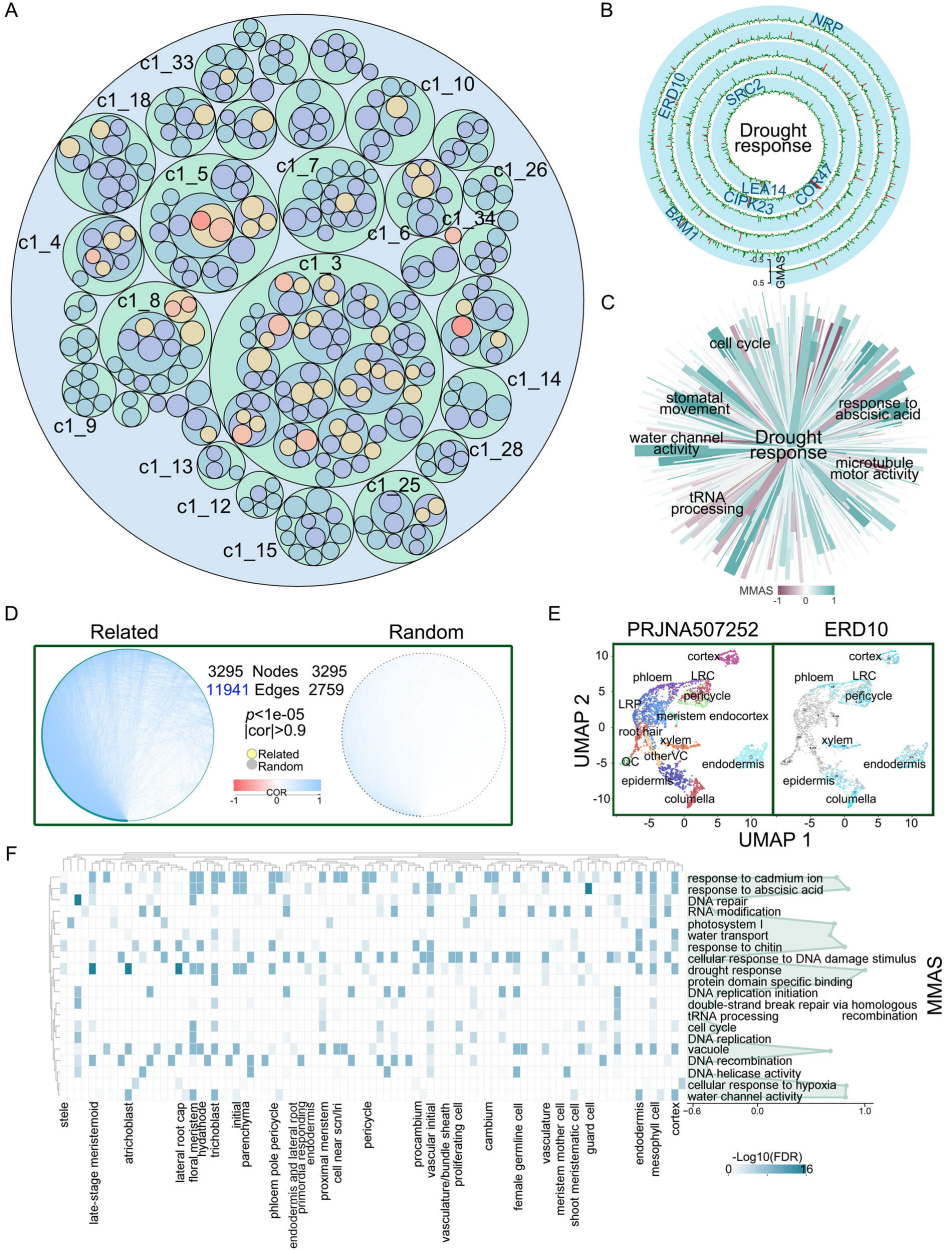
Characterization and analysis of co‐expression networks in plant drought response. A) The circular nesting diagram represents the hierarchical structure of the drought response network in *A. thaliana*, encompassing 21 420 genes and 455 modules. Each module is indicated by different colors, illustrating the organization of gene co‐expression. B) A spirochaete bar plot showing 588 drought‐responsive genes identified using the G‐MAD tool. Red highlights 339 known drought‐responsive genes, emphasizing their central role in drought response. C) Top functions associated with drought response were identified using the M‐MAD tool, with color depth reflecting the MMAS values of co‐functional networks related to drought response. D) Correlation network comparing the co‐functional networks of drought response versus random functions, derived from the 1001 Genomes Project. Network complexity and correlation strength are indicated by the number of edges and edge color intensity, respectively (*p* < 1e‐05). Nodes represent the number of related genes. E) UMAP plots of single‐cell transcriptome profiles from the PRJNA507252 dataset and *ERD10* dataset, colored by cell type. Notable cell types, including lateral root cap (LRC), lateral root primordium (LRP), quiescent center (QC), and other vascular cells (otherVC), are highlighted. F) Heatmap showing the enrichment of cell‐type‐specific marker genes in drought response‐related top functions (FET FDR < 0.01). A line graph on the right displays MMAS values between co‐functional networks and drought response, emphasizing functional relevance. See also Figures S1–S3 and Tables S2–S5, Supporting Information.

We identified gene modules of varying compactness, revealing a hierarchical structure of the drought response network in drought stress transcriptome data, which comprises 5 063 343 genes and 1599 modules (**Figure** **2**A; Figure S1 and Table S2, Supporting Information). Based on pan‐transcriptome data, we identified 588 genes highly associated with drought response (GO:0009414), including potential light stress‐regulated 3 (LSR3), asparagine‐rich protein 1 (NRP1), soybean gene regulated by cold‐2 (SRC2), cytidinediphosphate diacylglycerol synthase 5 (CDS5) (Figure 2B; Figure S2 and Table S3, Supporting Information). Among these, 339 are known drought response genes, including early responsive to dehydration 10 (*ERD10*), COLD‐REGULATED 47 (*COR47*), and BETA‐AMYLASE 1 (*BAM1*),^[^ ^17^ ^]^ indicating the reliability of our approach in recovering gene function information. At the protein level, we identified 11 864 genes associated with drought response using co‐fractionation mass spectrometry (CF‐MS),^[^ ^18^ ^]^ a high‐throughput technique for detecting protein interactions^[^ ^18^ ^]^ (Table S3, Supporting Information). To derive co‐functional and module‐to‐module association scores (MMASs), we employed M‐MAD to infer the association of genes closely linked to drought response, constructing a co‐functional drought response network. This network comprises 640 biological functions, where responses to abscisic acid and water channel activity were positively correlated with drought response; while, tRNA processing and cell cycle activities showed negative correlations (Figure 2C; Table S3, Supporting Information). Further validation was performed using a publicly available dataset from the *A. thaliana* populations in the 1001 Genome Project (PRJNA273563).^[^ ^13^ ^]^ Through correlation network analysis, we confirmed the conservation of co‐expression networks (Figure 2D; Table S4, Supporting Information). Single‐cell RNA sequencing (scRNA‐seq) data allowed us to identify the cell type‐specific transcriptomic structure within the drought response synergistic network. By leveraging these synergistic functions and performing enrichment analysis of cell type marker gene signatures from the scRNA‐seq dataset,^[^ ^14^ ^]^ we identified cell type‐specific network modules. It is well known that *ERD10* plays a key role in drought response.^[^ ^17a^ ^]^ We found that *ERD10* expression was primarily localized in the pericycle, endodermis, and cortex cells (PRJNA507252)^[^ ^19^ ^]^ (Figure 2E; Figure S3, Supporting Information). Using the cell‐specific localization of these known drought‐responsive genes, we further screened 588 high‐confidence candidate genes through enrichment analysis to obtain cell‐type‐specific drought‐responsive genes. We found that they were conservedly expressed in mesophyll, meristem, and vascular initiation cells, and partially expressed in quiescent center, upper protoderm, and lateral root primordium cells, indicating previously unrecognized common drought‐responsive cell characteristics. Unexpectedly, quiescent center cells demonstrated unique regulatory roles, suggesting their involvement in root architecture stabilization under drought conditions (Figure 2F; Table S5, Supporting Information).

### 2.3 Conserved Drought Response Functions Across Species

**Figure 3:**
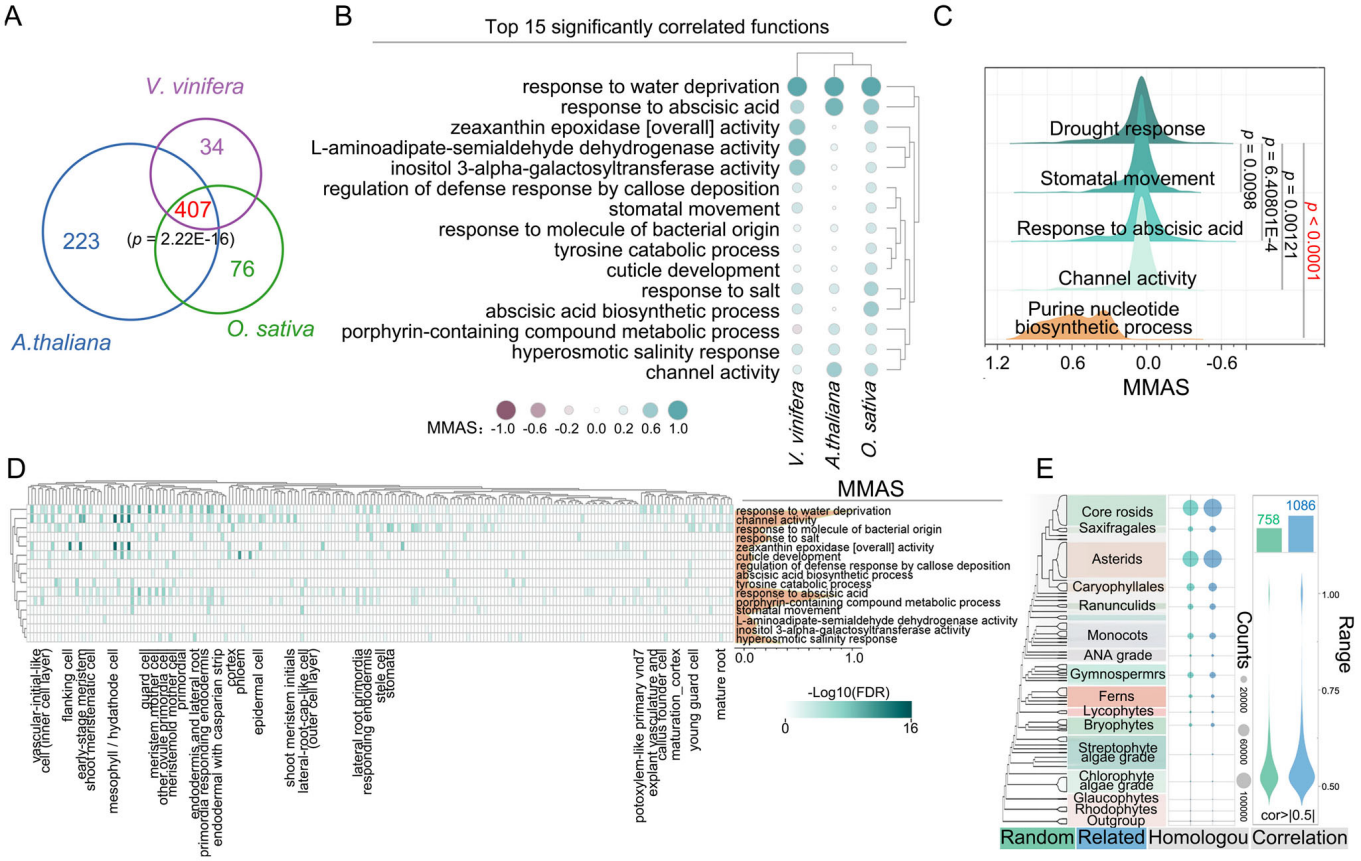
Preservation and cell‐type signatures in the co‐functional network of drought response. A) Venn diagram showing the overlap of co‐functions related to drought response across *V. vinifera*, *O. sativa*, and *A. thaliana* based on M‐MAD analysis. The significance of overlap was evaluated using a hypergeometric test (*p* = 2.22E‐16). B) Top 15 significantly conserved drought response functions across the three species based on M‐MAD analysis. Circle sizes represent the MMAS values, with red and green shades indicating negative and positive correlations, respectively. C) Cumulative distribution function plots of MMAS values for co‐functions related to drought response, stomatal movement, response to abscisic acid, channel activity, and purine nucleotide biosynthetic process. The K‐S test (one‐sided) was applied to assess statistical significance. D) Heatmap showing the enrichment of cell‐type‐specific marker genes in conserved drought response‐related functions (FET FDR < 0.01). The corresponding line graph shows the MMAS values between conserved co‐functions and drought response. E) Correlation networks of co‐functions or random functions (with the same number of related genes) constructed based on the OneKP project. Statistical significance (*p* < 0.01, LDS) was used to compare the complexity and correlation strength of the networks. The total number of conserved drought response functional orthologs (dots) is shown for each plant species. Violin plots display the correlation analysis results, with correlation values above 0.5 shown in green and blue for random and drought‐related groups, respectively. The plant phylogeny was adapted from previous studies.^[^ ^15^ ^]^ See also Tables S3–S5, Supporting Information.

To investigate the conserved functions of drought response across species, drought response functional networks of *V. vinifera*, *Oryza sativa*, and *A. thaliana*, representing diverse plant lineages, were constructed and the conservation of the co‐functional network was evaluated (*p* = 2.22E‐16) (**Figure** **3**A; Table S3, Supporting Information). Our analysis revealed several conserved drought response functions shared among these species, including 407 significantly correlated functions, such as stomatal movement, response to abscisic acid, and channel activity (Figure 3B; Table S3, Supporting Information). Notably, in contrast to the randomly distributed co‐functions of the purine nucleotide biosynthetic process, the comparison of stomatal movement, abscisic acid response, channel activity, and drought response co‐functions, based on MMAS scores, showed significant correlation (Kolmogorov–Smirnov test, *p* = 1E‐06) (Figure 3C).

To determine the cell type specificity of these conserved co‐functional networks in drought‐responsive species, we analyzed single‐cell transcriptome profiles of the top 15 co‐functions and identified marker genes across various cell types. The integration of co‐functions with cell type marker genes revealed distinct cell type specificity in drought responses (Figure 3D; Table S5, Supporting Information). For example, the drought response function was significantly enriched for marker genes in stomata, vascular‐initial‐like cells (inner cell layer), and meristem mother cells. This conservation is further corroborated by data from the OneKP project (E value < 1E‐05, amino acid identity > 60%), which spans all green plants dating back over 1 billion years (Figure 3E; Table S4, Supporting Information).

### 2.4 Genetic and Epigenetic Regulatory Factors Driving Drought Response

**Figure 4:**
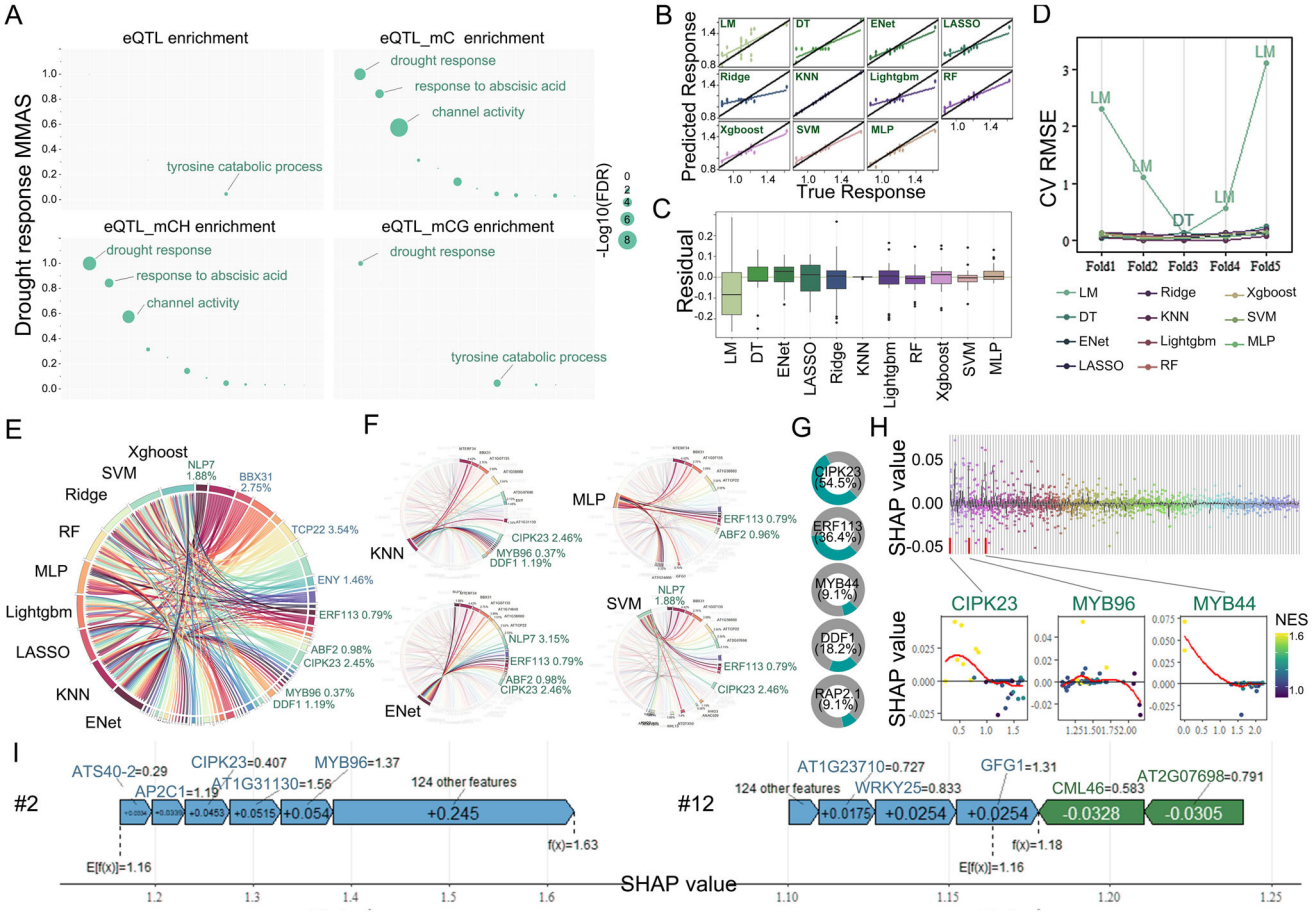
Genetic regulation of the co‐functional network of drought response. A) Scatter plot showing *cis*‐eQTL gene enrichment in the co‐functional network of drought response. MMAS values between co‐functions and drought response are plotted on the *Y*‐axis, with the size of the dots representing the FET enrichment significance of *cis*‐eQTL genes (FET FDR < 0.01). B) Scatter plot illustrating the relationship between predicted and true values for eleven different models (LM, DT, ENet, LASSO, Ridge, KNN, Lightgbm, RF, Xgboost, SVM, and MLP). Goodness of fit is represented by the distribution of points. C) Box plots showing the residual distributions of prediction errors for the eleven models to evaluate their performance. D) Dotted line plots of the root mean square error (RMSE) from cross‐validation (CV) across different models, assessing their generalization performance. E) Chord diagram showing the feature importance analysis based on SHAP values. The top 20 regulatory factors with SHAP values under each model are displayed, highlighting the relative importance of regulatory factors in multiple models. Green represents known drought response regulators; while, blue indicates potential regulators. F) Detailed visualization of feature contributions under KNN, MLP, ENet, and SVM models. The contribution ratios and connection relationships of regulatory factors are visualized using chord diagrams. G) Pie chart showing the proportion of the top 20 regulatory factors with SHAP values in each model, including CIPK23, ERF113, and MYB44, across all eleven models. H) Upper part: SHAP value distribution of regulatory factors under the optimal model KNN; lower part: SHAP dependency plot for CIPK23, MYB96, and MYB44, where each variable is plotted along a regression line. The red line indicates the mean SHAP value, and the normalized enrichment score (NES) is used for enrichment analysis of the gene expression dataset. I) Schematic diagram depicting the degree of influence of different regulatory factors in the random group on predicted results, highlighting their relative contributions. See also Figures S4 and S5 and Tables S5 and S6, Supporting Information.

Genetic variation (e.g., SNPs) and epigenetic modifications (e.g., DNA methylation) can alter gene expression in complex processes such as drought response. Here, we examined how genetic variation and epigenetic changes modulate the co‐functional network of drought response. To link methylation with expression differences, we mapped expression quantitative trait loci (eQTLs) to SNP data from the 1001 Genomes Project,^[^ ^20^ ^]^ identifying loci associated with gene expression. *Cis*‐eQTL analysis confirmed the association between gene expression levels and genotypes at local SNPs (within ± 1Mb of the gene) in the drought response co‐functional network. Using gene expression data and differentially methylated regions (100 bp; DMRs), we identified methylation‐dependent eQTLs and applied enrichment tests to integrate *cis*‐eQTL genes, methylation, and co‐functional networks. In the mC and mCH environments (FET *p* adj. < 0.05), methylation‐dependent *cis*‐eQTL genes were enriched in conserved drought response functions, with the strongest enrichment observed in response to abscisic acid, channel activity, and porphyrin‐containing compound metabolic processes (**Figure** **4**A; Table S5, Supporting Information).

To identify potential network regulators among co‐expressed genes, we constructed a causal network integrating genetic variation (SNPs),^[^ ^13^, ^20^ ^]^ gene expression, and known transcription factor‐target relationships (DAP‐seq).^[^ ^21^ ^]^ This network was built using eleven machine learning models: linear model (LM), decision tree (DT), elastic net (ENET), least absolute shrinkage and selection operator (LASSO), ridge regression (Ridge), k‐nearest neighbor (KNN), lightweight gradient boosting machine (LightGBM), random forest (RF), extreme gradient boosting (XGBoost), support vector machine (SVM), and multilayer perceptron (MLP).^[^ ^22^ ^]^ By projecting co‐functional genes into the causal network, we identified potential regulators, which were modular gene nodes surrounded by many co‐functional genes. A comparison of prediction performance and residual distributions across the eleven models showed that the KNN model provided the best prediction accuracy (Figure 4B,C; Figure S4 and Table S6, Supporting Information). Fivefold cross‐validation revealed that the KNN model had the lowest root mean square error (RMSE) (Figure 4D; Table S6, Supporting Information). After excluding the LM and DT models due to their higher RMSE, we evaluated the importance of regulatory factors within the co‐functional network across all models. The top‐ranked regulators included NIN Like Protein 7 (NLP7), which regulates nitrogen assimilation, osmosis, and ABA signaling to help plants adapt to water deficiency (1.88%);^[^ ^23^ ^]^ Calcineurin B‐like protein‐interacting protein kinase 23 (CIPK23), which modulates ion channels in the calcium signaling pathway, aiding ion homeostasis and osmotic balance under drought stress (2.45%);^[^ ^24^ ^]^ and other known drought response genes. In addition, B‐box domain‐containing protein 31 (BBX31) was found to enhance drought resistance through photomorphogenesis, antioxidant response, ABA signaling, and stomatal regulation;^[^ ^25^ ^]^ while Teosinte Branched1/Cycloidea/Proliferating Cell Factors 22 (TCP22) appeared to regulate plant cell growth, stomatal movement, and hormone signaling pathways in response to drought^[^ ^26^ ^]^ (Figure 4E; Table S6, Supporting Information). Further, the importance of regulatory factors in the co‐functional network was analyzed across different models (Figure 4F; Table S6, Supporting Information), revealing a high prevalence of CIPK23 and ERF113 (54.5% and 36.4%) among the top 20 regulatory factors (Figure 4G; Table S6, Supporting Information). Using Shapley additive explanation (SHAP) values, we confirmed that known drought response genes such as CIPK23 and MYB96 contributed most to the co‐functional network under the best‐performing KNN model (Figure 4H; Figure S5 and Table S6, Supporting Information). The SHAP value for a gene is computed based on the cooperative contribution of that gene in the context of all other genes in the model. The calculation considers all possible feature combinations and their corresponding marginal effects on the model's output. The SHAP value represents how much a particular gene's expression level influences the final prediction regarding its regulatory role in the drought response network. This contribution pattern was consistent across randomly selected samples (#2 and #12) (Figure 4I). In summary, these network analyses identified key regulatory drivers in the drought response co‐functional network.

### 2.5 Conserved Regulatory Factors Driving Drought Response Across Species

**Figure 5:**
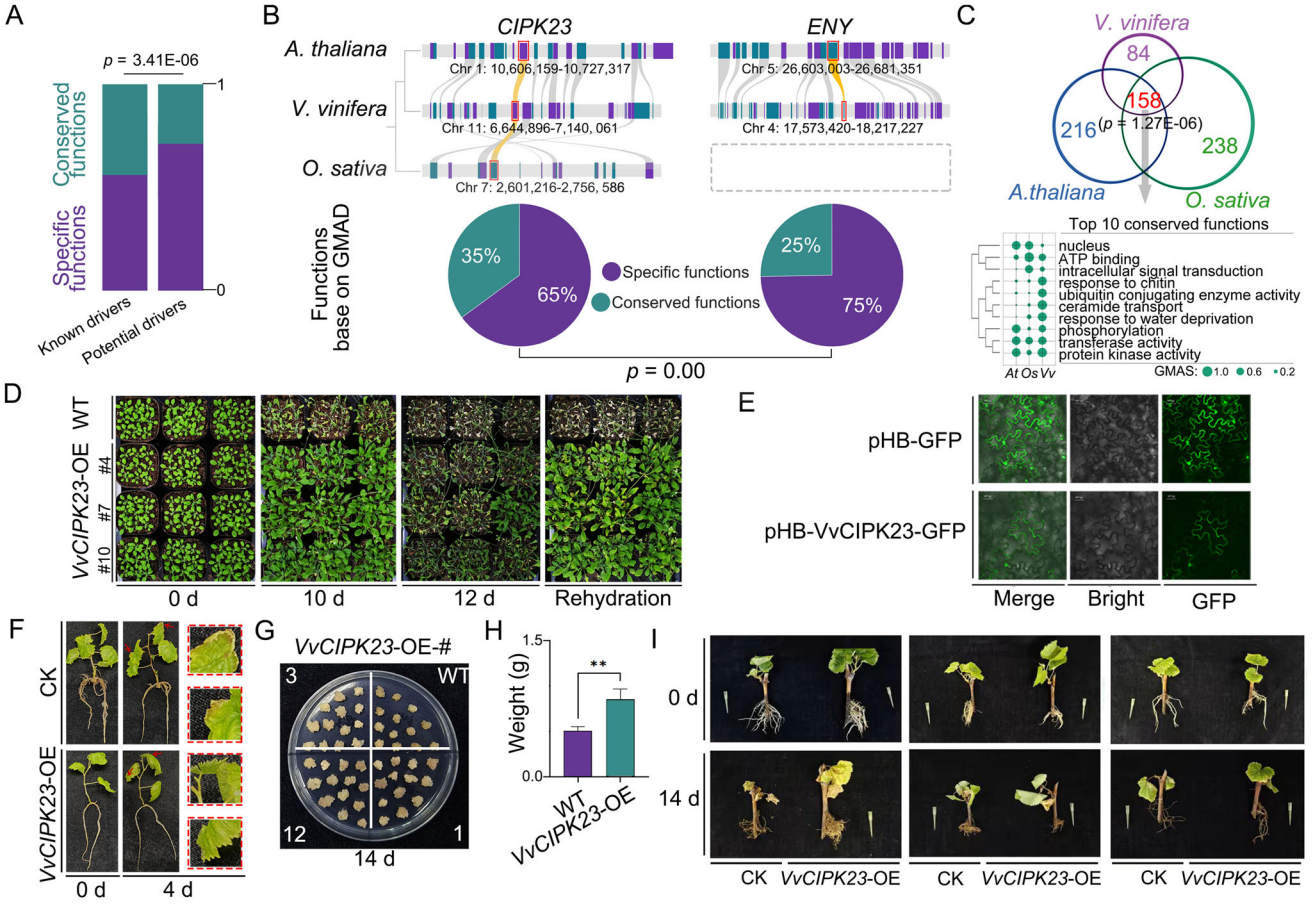
Conserved regulatory factors driving drought response across species. A) Statistical comparison of functional evolution in drought response drivers based on the co‐workflow and phylogenetic relationship developed in this study. Green represents conserved functions and purple indicates species‐specific functions. The significance was evaluated by the hypergeometric test (*p* = 3.41E‐06). B) Functional evolution of the known drought response driver CIPK23 and potential drought response factor ENY in *A. thaliana*, *V. vinifera*, and *O. sativa*. The upper panel shows the collinearity of these drivers across species, with gray boxes indicating the absence of collinear genes. The lower panel presents statistical results for conserved and species‐specific functions of the drivers (*p* = 0.00). C) Venn diagram showing the functional evolution of CIPK23 in *A. thaliana*, *V. vinifera*, and *O. sativa*, with significance assessed by the hypergeometric test (*p* = 1.27E‐06). The bottom panel lists the top ten conserved functions of CIPK23, with GMAS values indicated by the dots. D) Phenotypic analysis of VvCIPK23 overexpression (OE) in *A. thaliana* under drought stress. Plants were subjected to dehydration for 0, 10, and 12 days, followed by rehydration. Enhanced drought tolerance in VvCIPK23‐OE plants is visible, with better recovery post‐rehydration compared to wild type (WT). E) Subcellular localization of the VvCIPK23‐GFP fusion protein in tobacco cells. GFP fluorescence indicates that the VvCIPK23 fusion protein localizes to the nucleus and cytoplasm, similar to AtCIPK23. Scale bar: 30 µm. F) Transient overexpression of VvCIPK23 in grape tissue culture seedlings enhances drought tolerance. Four‐week‐old seedlings were submerged in Agrobacterium solutions containing either the empty vector (CK) or the recombinant plasmid, followed by hydroponic culture and drought simulation (200 mm mannitol). The red arrows indicate browning and curling of leaf edges, with magnified views shown in the red boxes. G,H) Overexpression of VvCIPK23 in grape callus enhances drought tolerance. After 14 days on 200 mm mannitol medium, callus clusters from VvCIPK23‐OE plants exhibited less browning compared to wild‐type clusters. The fresh weight of ten callus clusters per group was measured, showing a significant difference between WT and transgenic calluses (*p* < 0.01; Student's *t*‐test) (*n* = 10). Error bars represent standard errors. I) Overexpression of VvCIPK23 in grape green cuttings via *A. rhizogenes*‐mediated transformation. After 12 days of drought stress, control cuttings exhibited severe wilting; while, the VvCIPK23‐overexpressing plants showed significantly less wilting, indicating improved drought tolerance. See also Figure S6 and Table S7, Supporting Information.

By integrating co‐expression and causal networks, we identified *CIPK23*, *ERF113*, and *DDF1* as key contributors to the co‐functional network involved in the plant drought response. These regulatory factors exhibited strong associations with drought‐related pathways and processes. Using the workflows developed in this study, we explored the functional evolution of these drivers in various plant species, including the horticultural crop *V. vinifera*, the monocot *O. sativa*, and the dicot *A. thaliana*. Compared to other potential drought‐response drivers such as *ENY* and *ERF74*, known regulators such as *CIPK23* ^[^ ^24^ ^]^ and *ZAT10* ^[^ ^27^ ^]^ were found to be more conserved across species, displaying less species‐specific functional variability (**Figure** **5**A,B; Table S7, Supporting Information).

Notably, *CIPK23*, a critical protein kinase,^[^ ^24^ ^]^ has been conserved in its role in mediating plant responses to multiple stressors, with a particular emphasis on drought tolerance (Figure 5C; Figure S6A–E and Table S7, Supporting Information). The conserved role of CIPK23 in ion homeostasis and ABA signaling underpins its potential as a universal target for crop improvement. To further validate *CIPK23* as a conserved drought‐response regulator, we overexpressed the *V. vinifera* ortholog of *CIPK23* (*VvCIPK23*) in *A. thaliana* (Figure 5D,E). The overexpression of *VvCIPK23* in *A. thaliana* resulted in significantly enhanced drought tolerance, supporting its functional conservation. In addition, we transiently overexpressed *VvCIPK23* in *V. vinifera* plants (Figure 5F; Figure S6F,G, Supporting Information), stably overexpressed it in callus tissues (Figure 5G,H; Figure S6H,I, Supporting Information), and transformed it into grape green cuttings via the Agrobacterium‐mediated hairy root transformation system (Figure 5I). These overexpressing plants exhibited improved resistance to multiple abiotic stresses, particularly drought, highlighting the potential of *CIPK23* as a key target for enhancing stress tolerance across species.

### 2.6 Multi‐Omics and AlphaFold 3 Identify Core Protein Interactions Driving Plant Drought Resistance

**Figure 6:**
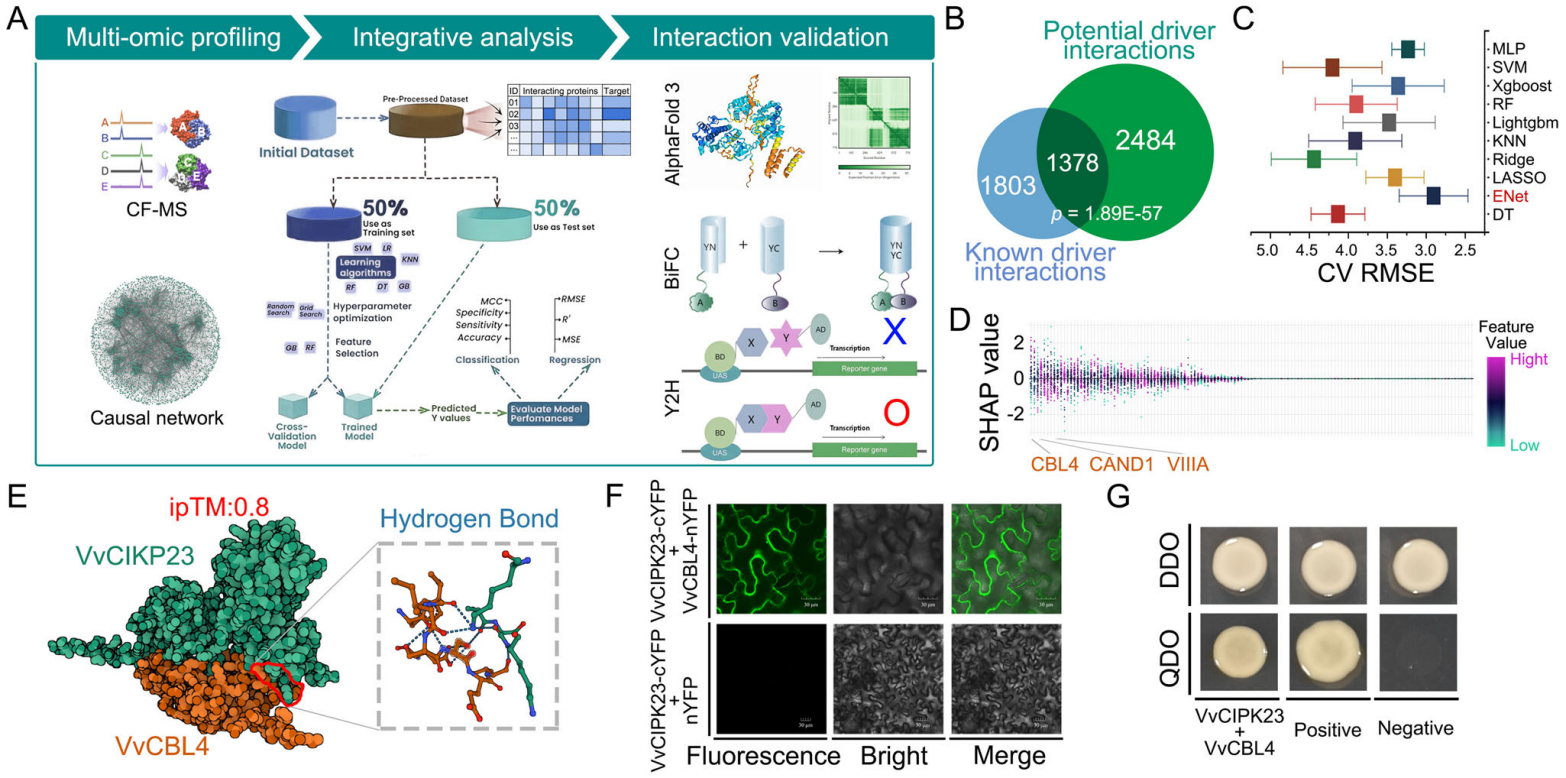
Core protein interactions driving plant drought resistance. A) Schematic overview of the workflow for analyzing protein interactions with drought response drivers. Proteins binding to drought response drivers were identified using CF‐MS and mapped to a causal network for drought response. Eleven machine learning methods were employed to rank proteins interacting with the drought response drivers. Protein–protein interactions were validated using sequencing results from AlphaFold3, BiFC, and Y2H methods. B) Venn diagram illustrating the distribution of known versus potential drought response driver binding proteins. The significance of overlap was evaluated using the hypergeometric test (*p* = 1.89E‐57). C) Box plots displaying the RMSE values from cross‐validation (CV) to evaluate the generalization performance of different machine learning models (ENet, MLP, Xgboost, SVM, etc.). D) Scatter plot showing the SHAP value‐based ranking of proteins interacting with CIPK23 under the optimal model (ENet), highlighting the importance of specific protein interactions. E) Verification of the interaction between VvCIPK23 and VvCBL4 using AlphaFold3. The red box highlights the hydrogen bonding region between the two proteins, with the interface predicted template modeling (ipTM) score indicating the confidence level of the interaction (ipTM = 0.8). Values above 0.8 represent high‐confidence predictions.^[^ ^41^ ^]^ F) BiFC assay confirming the interaction between *VvCIPK23* and *VvCBL4*. *VvCIPK23* was fused with the C‐terminal YFP tag, and VvCBL4 was fused with the N‐terminal YFP tag. Co‐injection into tobacco leaves, followed by 48 h of dark incubation showed positive fluorescence signals, indicating protein interaction. Scale bar = 30 µm. G) Yeast two‐hybrid (Y2H) assay confirming the interaction between *VvCIPK23* and *VvCBL4*. The PGBKT7‐VvCIPK23 and PGADT7‐VvCBL4 vectors were co‐transformed into yeast strain Y2H, and protein interactions were assessed on selective media (QDO). Positive and negative controls are included as indicated. See also Figure S7 and Tables S8–S10, Supporting Information.

Integrating AlphaFold 3′s protein–protein interaction predictions,^[^ ^28^ ^]^ CF‐MS,^[^ ^18^ ^]^ and causal network analysis (**Figure** **6**A; Table S8, Supporting Information), we observed that known drought response drivers, such as CIPK23^[^ ^24^ ^]^ and MAPK3,^[^ ^29^ ^]^ interact with fewer types of proteins and exhibit higher levels of conservation across species compared to potential drought response drivers such as WRKY40 and MTERF34 (Figure 6B). Specifically, CIPK23 was predicted to interact with 130 different proteins, with the strongest interaction identified between CIPK23 and CBL4 (Figure 6C,D; Table S9, Supporting Information). This interaction plays a pivotal role in the CPL‐CIPK signaling pathway, which is critical for plant stress responses.

To validate the predicted interaction between CIPK23 and CBL4 in *V. vinifera*, we employed a combination of in silico and experimental approaches. The AlphaFold 3 predictions were further corroborated using bimolecular fluorescence complementation (BiFC) and yeast two‐hybrid (Y2H) assays (Figure 6E–G; Figure S7, Supporting Information), confirming the physical interaction between CIPK23 and CBL4 in grapevine. These results provide compelling evidence for the conserved function of the CIPK23‐CBL4 interaction in plant drought response, enhancing our understanding of how the CPL‐CIPK signaling system contributes to drought tolerance across diverse species.

## 3 Discussion

This study provides a comprehensive multi‐omics integration and network analysis of plant drought responses across different species, incorporating transcriptomics, proteomics, and single‐cell RNA sequencing (scRNA‐seq) data. Through the analysis of nearly 30 000 raw data samples and a multi‐stage network analysis, we constructed a hierarchical gene regulatory network specific to drought response at the cellular level. Using the workflow we developed, 588 genes were identified that were significantly associated with drought response, including potential light stress‐regulated 3 (LSR3), asparagine‐rich protein 1 (NRP1), soybean gene regulated by cold‐2 (SRC2) cytidinediphosphate diacylglycerol synthase 5 (CDS5), and well‐known regulators such as *ERD10* ^[^ ^17a^ ^]^ and *BAM1*.^[^ ^17c^ ^]^ In addition, genetic and epigenetic factors driving these drought responses were explored, with key regulators such as *CIPK23*, *ERF113*, and *DDF1* emerging as crucial contributors to drought tolerance. The role of CIPK23 in drought tolerance was experimentally validated across multiple species, emphasizing the conserved nature of these regulatory networks. Moreover, through AlphaFold 3 and CF‐MS analysis, key protein interactions, such as the *CIPK23*‐*CBL4* complex, were identified, offering deeper insights into the molecular mechanisms underlying drought resistance.

Previous studies have emphasized the role of *ERD10* ^[^ ^17a^ ^]^ in dehydration stress, consistent with our results confirming its expression in pericycle and endodermis cells. Similarly, *BAM1*’s involvement in starch breakdown during drought conditions has been well‐documented,^[^ ^30^ ^]^ and its identification in our study further supports its role under stress. The co‐functional network we constructed corroborates findings from other multi‐omics studies, especially in areas such as abscisic acid (ABA) signaling, stomatal regulation, and osmotic balance during drought. Our results confirm these functions and extend them by identifying conserved modules across species. Recent studies in *A. thaliana* have demonstrated the critical role of ABA in drought responses,^[^ ^31^ ^]^ which aligns with our findings of ABA‐related functions being conserved in *V. vinifera* and *O. sativa*. These results highlight the robustness of our multi‐omics approach in capturing both species‐specific and conserved mechanisms. The identification of CIPK23 as a key regulator mirrors past findings on its role in ion homeostasis and ABA signaling pathways during water deficiency.^[^ ^24^ ^]^ Previous research has shown that stress response genes such as CIPK23 are conserved across species, and our validation of CIPK23's role in enhancing drought resistance in *A. thaliana* and *V. vinifera* reinforces its importance in mediating responses to drought and other abiotic stresses.

A particularly unexpected result of this study was the identification of novel cell‐type‐specific functions in the drought response network. While many drought‐responsive genes, such as *ERD10* ^[^ ^17a^ ^]^ and *COR47*,^[^ ^17e^ ^]^ have well‐established roles, their specific expression patterns in pericycle, endodermis, and cortex cells were previously unreported. Our scRNA‐seq analysis revealed that these genes not only exhibit conserved functions but also possess cell‐type‐specific roles, providing new insights into tissue‐specific responses to drought stress. This suggests a more nuanced regulatory mechanism in which specific cell types contribute uniquely to drought adaptation, an aspect that has been underexplored in previous studies. In addition, the identification of drought‐responsive features in quiescent center and lateral root primordium marker genes was an unexpected finding. These regions have not typically been associated with drought responses; yet, our data suggest that these cell types may play important roles in maintaining drought resilience, likely through root structure stabilization and water uptake under stress conditions. This discovery could open new avenues for research into root‐specific drought adaptation strategies.

Our results significantly contribute to the broader theoretical framework of plant stress response and adaptation. Traditional models of drought response, which focus on ABA signaling and stomatal regulation, are expanded by our identification of novel gene regulatory networks that integrate genetic and epigenetic factors. The inclusion of *cis*‐eQTL and methylation data in the co‐functional network provides a more comprehensive view of how genetic variation and epigenetic modifications influence drought responses. This aligns with modern theories of stress adaptation, which propose that phenotypic plasticity in response to environmental stress is mediated through complex gene‐environment interactions.^[^ ^32^ ^]^ Our causal network analysis supports the emerging concept of modular gene regulation in stress responses. By identifying key regulators such as CIPK23 and ERF113, which are modularly connected to numerous co‐functional genes, we have illustrated how drought tolerance is orchestrated by a small number of master regulators driving broad transcriptional changes. This hierarchical regulatory model fits well with existing theories in network biology, where a few central hubs exert control over large subnetworks, ensuring coordinated responses to stress.^[^ ^33^ ^]^ Further, the conservation of these regulatory factors across species, demonstrated through our cross‐species network analysis, supports the idea of evolutionarily conserved mechanisms in stress adaptation. This is particularly relevant in evolutionary biology, where drought response is recognized as a key adaptive trait in plant species. Our findings underscore the importance of conserved regulatory networks in enabling plant survival under extreme environmental conditions, reinforcing evolutionary theories that emphasize the role of conserved molecular mechanisms in stress adaptation.

Despite the strengths of our multi‐omics integration and network analysis, several limitations should be acknowledged. One limitation is the potential for biases introduced by differences in experimental design, data quality, and sample heterogeneity. Although we employed rigorous normalization and validation procedures to mitigate these biases, unaccounted variability may still exist, particularly in cross‐species comparisons where differences in experimental conditions could obscure true biological conservation. Another limitation is the focus on transcriptomic and proteomic data, with limited integration of metabolomic datasets. Metabolomic data could provide additional insights into the biochemical pathways activated during drought stress, potentially revealing further conserved functions or regulators. Future studies should aim to incorporate metabolomics into the multi‐omics framework for a more comprehensive understanding of drought responses. While the identification of key regulators such as CIPK23 and ERF113 is promising, experimental validation across a broader range of species and environmental conditions is needed to fully confirm their roles in drought tolerance. Our experimental validation was limited to a few model species, and further research is necessary to assess the applicability of these findings to other crops or wild plant species. In addition, potential trade‐offs between drought resistance and other traits, such as growth or reproductive fitness, should be explored in future studies. Last, the resolution of our scRNA‐seq data posed some challenges. The identification of marker genes for specific cell types was constrained by the resolution of available datasets. More refined single‐cell techniques will be required to capture the full complexity of cellular responses to drought. Advances in scRNA‐seq technology, such as increased sequencing depth and improved cell type identification, will be crucial for furthering our understanding of cell‐type‐specific drought responses. These findings emphasize the potential of leveraging conserved regulatory networks, such as CIPK23‐mediated pathways, for breeding drought‐resilient crops. However, sample heterogeneity across datasets remains a challenge, warranting future studies with more standardized experimental conditions.

## 4 Conclusion

This study presents a thorough and innovative integration of multi‐omics datasets to elucidate the regulatory networks driving plant responses to drought, with a particular focus on cell‐type‐specific and conserved functions. It offers both expected confirmations of known pathways, such as ABA signaling and stomatal regulation, and new insights, such as the negative correlation of metabolic processes with drought tolerance and the identification of non‐canonical regulatory factors. The discovery of highly conserved drought response mechanisms across species not only underscores the evolutionary importance of these networks but also offers promising targets for crop improvement efforts. This study provides a roadmap for translating molecular insights into practical solutions, paving the way for developing resilient crops to address global water scarcity challenges. However, future research should focus on expanding the range of species studied, further validating the computational predictions in vivo and delving deeper into the functional significance of cell‐type‐specific drought responses.

## 5 Experimental Section

### Plant Materials and Growth Conditions

The plant materials used in this study were provided by the Research Group on the Molecular Mechanism of Fruit Tree Resistance and Stress‐Resistant Genetic Breeding at the School of Agriculture and Biology, Shanghai Jiao Tong University. Callus cultures of Thompson Seedless grapevines were maintained at 25 °C in complete darkness. Tissue culture seedlings of Thompson Seedless were cultivated under sterile conditions at 25 °C with a 16‐h light and 8‐h dark photoperiod. *A. thaliana* plants were grown at 22 °C with the same 16‐h light and 8‐h dark cycle; while, Nicotiana benthamiana (tobacco) plants were cultivated at 25 °C with identical photoperiodic conditions.

### GeneBridge Analysis

The selection of the 30 000 data samples was guided by the completeness of metadata, coverage of drought response conditions, and availability of scRNA‐seq datasets for cell‐specific analysis. The integration of data from different tissues was performed carefully by considering tissue‐specific regulatory mechanisms that were known to exist. The integration strategy involved selecting species that were closely related in terms of their evolutionary lineage and drought stress responses. The PEER tool was utilized to preprocess the data and eliminate potential confounding variables.^[^ ^34^ ^]^ Following this step, the data were transferred to the GeneBridge toolkit for further analysis. In the GeneBridge toolset, ontology terms, biological pathways, and knowledge‐based gene sets from diverse resources are referred to as “modules;” however, only ontology terms were used in this study. To infer possible gene functions, the G‐MAD tool was employed within the GeneBridge toolkit, which utilizes expression data from large‐scale cohorts. To assess the associations between relevant genes and biological modules, the correlation Adjusted MEan RAnk (CAMERA) gene set test, a competitive gene set testing technique integrated in G‐MAD, accounted for inter‐gene correlations.^[^ ^18^ ^]^ A binding score of 1 or ‐1, based on the direction of enrichment, was assigned to gene‐module interactions with enrichment *p*‐values that remained significant after multiple testing adjustments. Otherwise, a binding score of 0 was assigned.

Following a meta‐analysis of the datasets, the average binding scores were weighted by the inter‐gene correlation coefficient within modules (¯p) and sample size to generate gene‐module association scores. Cross‐species transcriptome compendia were used to identify linkages between modules through M‐MAD from the GeneBridge toolbox. The G‐MAD results for each module against all genes were used to determine the relationships between genes and modules. Gene‐level data collected using the CAMERA technique were applied to calculate enrichment scores for all genes against the target modules. Based on Bonferroni‐corrected thresholds, the cross‐module *p*‐values were condensed and adjusted to 1, 0, or −1. A meta‐analysis was then performed across all datasets to produce module–module association scores (MMAS).

The module association network was constructed by analyzing the complete expression datasets; while, previously published gene annotations were used to build the module similarity network. This module association network enabled the identification of novel biological linkages between modules that may not have been previously documented in the literature.

### Expression QTL Mapping

Transcript expression and SNP genotype data were integrated from the 1001 Genomes Project to identify expression quantitative trait loci (eQTLs). The SNP matrix, consisting of 1 673 530 variants, was used as the genotype data for eQTL analysis. For epigenetic eQTL (eQTL^epi^), differentially methylated bins (CG‐, CH‐, and C‐DMBs) were used as genotypes, with the same sample correlation matrices as those calculated for eQTL analysis.^[^ ^20^ ^]^ Significant correlations were identified by applying the Benjamini & Hochberg (BH) correction, selecting *p*‐values less than 0.05.

eQTLs were classified as *cis* if the association peak was located within 1 Mb on either side of the gene's exon boundary. Conversely, they were defined as *trans* if the peak was located at least 5 Mb away from the exon boundary. Variants with association *p*‐values < 2.46 × 10^4^ (corresponding to a 1% false discovery rate, FDR) within the *cis* region were considered significant. For *trans* eQTLs, a more stringent threshold of BH‐corrected *p* < 1.51 × 10^13^ was applied. Genes regulated by *cis* eQTLs were referred to as *cis* eQTL genes.

### Causal Network Inference

To infer regulatory relationships among genes, DAP‐seq transcription factor (TF)‐target interactions and eQTL analysis were utilized as prior information. In the causal network, TFs served as parent nodes to their target genes; while, target genes could not serve as parent nodes to their respective TFs. The causal network was constructed by integrating TF‐target interactions with eQTL‐based genetic relationships.

To predict driver genes regulating downstream nodes, co‐functional genes were mapped onto the constructed causal network. Nine widely‐used machine learning and deep learning models—Linear Model (LM), Decision Tree (DT), Elastic Net (ENET), K‐Nearest Neighbors (KNN), LightGBM, Random Forest (RF), XGBoost, Support Vector Machine (SVM), and Multi‐Layer Perceptron (MLP)—were employed to screen for key driver genes. These key genes were identified as nodes enriched in the co‐functional gene network neighborhood.^[^ ^22^ ^]^

A fivefold cross‐validation scheme was used to evaluate model performance, with Bayesian optimization employed as a surrogate model for the acquisition function. Finally, model interpretability was enhanced through Shapley additive explanations (SHAP)^[^ ^35^ ^]^ (https://github.com/slundberg/shap), which were used to visualize prediction results and explain the selection criteria for the optimal model.

### Single‐Cell RNA‐Seq Analysis

To determine the cell‐type specificity of co‐functional modules, Fisher's Exact Test (FET) was employed from the R package clusterProfiler (v3.18).^[^ ^36^ ^]^ The test was used to assess the overlap between cell‐type marker genes and co‐functional module genes, with enrichment *p*‐values calculated based on deviations from the null hypothesis. Adjusted *p*‐values (Padj) were computed using the Benjamini–Hochberg (BH) method for multiple testing corrections. Finally, non‐linear dimensionality reduction and cluster visualization were performed using the UMAP algorithm.

### Transcriptome Analysis

Raw RNA reads were filtered and trimmed using Trimmomatic (v0.39),^[^ ^37^ ^]^ and their quality was assessed with FastQC (v0.11.9).^[^ ^38^ ^]^ Clean, high‐quality reads were then mapped to the draft reference genome using HISAT2. Gene expression values, measured in transcripts per million (TPM), were calculated with FeatureCounts (v1.6.3).^[^ ^39^ ^]^ Normalization of gene expression levels (BaseMean) across samples was performed using the DESeq2 package.^[^ ^40^ ^]^ Differentially expressed genes (DEGs) were identified with a significance threshold of Padj (adjusted *p*‐value) < 0.05 for each comparison group.

### GO Enrichment Analysis

Gene ontology (GO) enrichment analysis of single‐copy orthologs of the expressed proteins (EPs) was conducted using the GOseq R package (release 3.15). Differentially expressed genes (DEGs) were considered significantly enriched for GO terms if the adjusted *p*‐value was below 0.05. To further categorize and visualize the functional distribution of gene functions, GO annotations were analyzed using WEGO software.

### Construction and Visualization of the Correlation Network

The R package imsbInfer from GitHub was used to create the correlation network. Using Pearson's rank correlation measure, correlations were calculated; positive correlations were shown by red edges; while, negative correlations were indicated by blue edges.

### Generation of DNA Constructs

The coding sequence (CDS) of *VvCIPK23* was amplified via PCR from Thompson Seedless leaf cDNA using gene‐specific primers (pHB‐VqCIPK23‐F/R) (Table S10, Supporting Information). The resulting amplicon was inserted into the pHB vector under the control of the CaMV 35S promoter for gene expression. This construct was used for the genetic transformation of *A. thaliana*, grapevine callus, and the transient transformation of grapevine tissue culture seedlings.

To create the *VvCIPK23*‐GFP fusion protein, the full‐length CDS of *VvCIPK23* (excluding the stop codon) was amplified using specific primers and inserted into the pHB‐GFP (green fluorescent protein) vector, generating the expression vector pHB‐*VvCIPK23*‐GFP. This vector was used for subcellular localization assays.

For the yeast two‐hybrid (Y2H) assay, the full‐length CDS of *VvCIPK23* was cloned into the pGBKT7 (BD) vector to generate the BD‐*VvCIPK23* bait; while, the CDS of *VvCBL4* was cloned into the pGADT7 (AD) vector to create the AD‐*VvCBL4* prey.

In the bimolecular fluorescence complementation (BiFC) assay, the full‐length CDS of *VvCIPK23* and *VvCBL4* (without stop codons) was inserted into the pXY‐106‐nYFP and pXY‐104‐cYFP vectors, respectively, for interaction studies.

### *Arabidopsis* Genetic Transformation

Wild‐type *A. thaliana* seeds were surface‐sterilized with a 2% sodium hypochlorite solution for 10 min and evenly sown onto 1/2 Murashige and Skoog (MS) medium. After 7–10 days of growth under long‐day conditions, the seedlings were transferred to nutrient soil. After 30 days, healthy *Arabidopsis* plants with robust growth and multiple inflorescences were selected. To prepare for transformation, the siliques were removed 1 day prior to infection.

*Agrobacterium tumefaciens* carrying the pHB‐*VvCIPK23* construct was cultured to an OD600 of 1.0. The bacterial suspension was then diluted to an OD600 of 0.5 using 1/2 MS infection solution containing 5% sucrose and 0.05% Silwet‐77. The *Arabidopsis* inflorescences were immersed in the bacterial solution for 1 min, and the plants were kept in the dark for 24 h. The next day, the plants were returned to normal light conditions for continued growth. The infection process was repeated after 7–10 days, once new inflorescences had developed.

### Grapevine Callus Transformation

*Agrobacterium tumefaciens* containing the pHB‐*VvCIPK23* construct for grapevine callus transformation was cultured to an OD600 of 1.0. The culture was diluted to an OD600 of 0.5 using MS infection solution supplemented with 3% sucrose and 0.1 mm acetosyringone (AS), and this suspension was used to infect seedless white grape callus. The infected callus clusters were co‐cultivated on 1/2 MS solid medium containing 3% sucrose and 0.1 mm AS for 3 days. Following co‐cultivation, the callus was transferred to a selection medium containing 10 mg L^−1^ picloram (Pir), 2.2 g L^−1^ thidiazuron (TDZ), 50 mg L^−1^ hygromycin (Hyg), and 200 mg L^−1^ timentin (TMT) for further growth. The transgenic callus clusters were subsequently identified at the RNA level using RT‐qPCR.

### Grapevine Whole‐Plant Transient Transformation

*Agrobacterium tumefaciens* containing the pHB‐*VvCIPK23* construct for grapevine whole‐plant transient transformation was cultured to an OD600 of 1.0. The bacterial suspension was then diluted to an OD600 of 0.5–0.6 using MES buffer (10 mm MES, 10 mm MgCl2, and 100 µm acetosyringone). Five‐week‐old tissue culture seedlings, exhibiting uniform growth vigor, were removed from the medium, and the basal leaves were trimmed. A vacuum infiltration method was employed for the transient transformation. The seedlings were inverted into a glass jar containing the *Agrobacterium* suspension and subjected to vacuum infiltration at −0.1 Mpa for 10 min, repeated twice. During infiltration, the leaves became semi‐transparent. The infiltrated seedlings were then transplanted into soil with the roots secured, and the plants were covered with a plastic bottle to maintain high humidity. After co‐cultivation under low‐light conditions at room temperature for 4 days, samples were collected for subsequent experiments.

### *Agrobacterium Rhizogenes*‐Mediated Transformation of Grape Hairy Roots

*A. Rhizogenes* strain MSU440, carrying either the empty vector or the recombinant vector pNMGFP‐*VvCIPK23*, was cultured in liquid medium until the bacterial suspension reached an OD600 of 1.0. The bacterial cells were then harvested and resuspended in MES buffer to an OD600 of 0.6–0.7. Diploid green branches of “Shen Ai” were cut into 8–10 cm segments, each containing a single bud. The cuttings were immersed in the bacterial suspension and co‐cultivated in darkness at room temperature for 24 h. Following co‐cultivation, the cuttings were transferred to moist, nutrient‐rich soil mixed with vermiculite for further growth. To maintain humidity and promote root formation, tray covers were placed over the cuttings for the first 2 weeks. After 5–6 weeks, root development was assessed, and cuttings with uniform root growth were selected for subsequent experiments.

### Subcellular Localization Experiment

The *Agrobacterium* liquid culture containing the pHB‐*VvCIPK23*‐GFP construct for subcellular localization was grown to an OD600 of 1.0. The bacterial cells were then harvested and resuspended in MES buffer to adjust the OD600 to 0.4–0.5. The bacterial suspension was kept in the dark for 2 h before being used to infiltrate 4–6‐week‐old wild‐type tobacco plants. Following injection, the plants were incubated in the dark for 48 h. Samples were collected from the injection sites using a hole punch, and the subcellular localization of the pHB‐*VvCIPK23*‐GFP fusion protein was observed using a confocal laser scanning microscope (FV300) at an excitation wavelength of 488 nm to detect GFP fluorescence.

### Yeast Two‐Hybrid (Y2H) Assay

The pGBKT7‐VvCIPK23 and pGADT7 vectors were co‐transformed into Y2H Gold yeast competent cells for self‐activation testing. The transformed yeast cells were first cultured on SD/‐Trp/‐Leu double dropout solid medium for 2–3 days to obtain single clones. These clones were then transferred to SD/‐Trp/‐Leu/‐His/‐Ade quadruple dropout solid medium for 4–5 days to observe whether any yeast colonies grew.

After confirming that pGBKT7‐VvCIPK39 does not autonomously activate the expression of reporter genes in yeast, the pGBKT7‐VvCIPK39 vector was co‐transformed with the target protein plasmid pGADT7‐VvCBL4 into Y2H Gold yeast competent cells. Similar to the previous method, the single clones obtained on double dropout solid medium were transferred to quadruple dropout solid medium for 4–5 days to observe whether yeast colonies grew.

### Bimolecular Fluorescence Complementation Assay (BiFC)

The constructed BiFC vectors pXY‐106‐VvCIPK23‐nYFP and pXY‐104‐VvCBL4‐cYFP were separately transformed into agrobacterium tumefaciens containing the p19 plasmid. After obtaining positive single clones, the liquid cultures were grown to OD_600_ = 1.0. The bacterial cells were collected and resuspended in MES buffer to adjust the OD_600_ to 0.5–0.6. The two bacterial suspensions were mixed at 1:1 ratio and incubated in the dark for 2 h. The mixture was then injected into tobacco leaves as previously described. After 48 h, the yellow fluorescent protein (YFP) signal was observed using a confocal laser scanning microscope.

### Statistical Analysis

The experimental data were analyzed using Origin Pro 2024 software (OriginLab Corporation, Northampton, Massachusetts, USA). The significance of differences between groups was determined using least significant difference tests, and the thresholds for statistical significance were 0.05 and 0.01.

## Conflict of Interest

The authors declare no conflict of interest.

## Author Contributions

M.L. and Y.X. contributed equally to this work. M.L. planned and designed the research. M.L. and Y.S. analyzed the data. M.L. and Y.X. wrote the original manuscript. Y.X., D.F., and L.W. used the method to integrate plant data. Y.X., Z.Z., and C.C. reviewed and edited the manuscript. M.L., J.L., and J.H. revised the manuscript. C.M. supervised the research. All authors read and approved the final manuscript.

## Supporting information



Supporting Information

Supplemental Tables

## Data Availability

The data that support the findings of this study are available in the Supporting Information of this article.
