# Supplementary material for: Hierarchical Regulatory Networks Reveal Conserved Drivers of Plant Drought Response at the Cell‐Type Level
Source: Adv Sci (Weinh). 2025 Mar 16;12(18):2415106. doi: 10.1002/advs.202415106 (PMC12079547; doi:10.1002/advs.202415106)
Supplement: Supplementary file 1 — Supporting Information [file ADVS-12-2415106-s002.docx]

Supporting Information

**Hierarchical Regulatory Networks Reveal Conserved Drivers of Plant Drought Response at the Cell-Type Level**

*Moyang Liu, Yuanyuan Xu, Yue Song, Dongying Fan, Junpeng Li, Zhen Zhang, Lujia Wang, Juan He, Cheng Chen, and Chao Ma**

**Supplementary Figures**


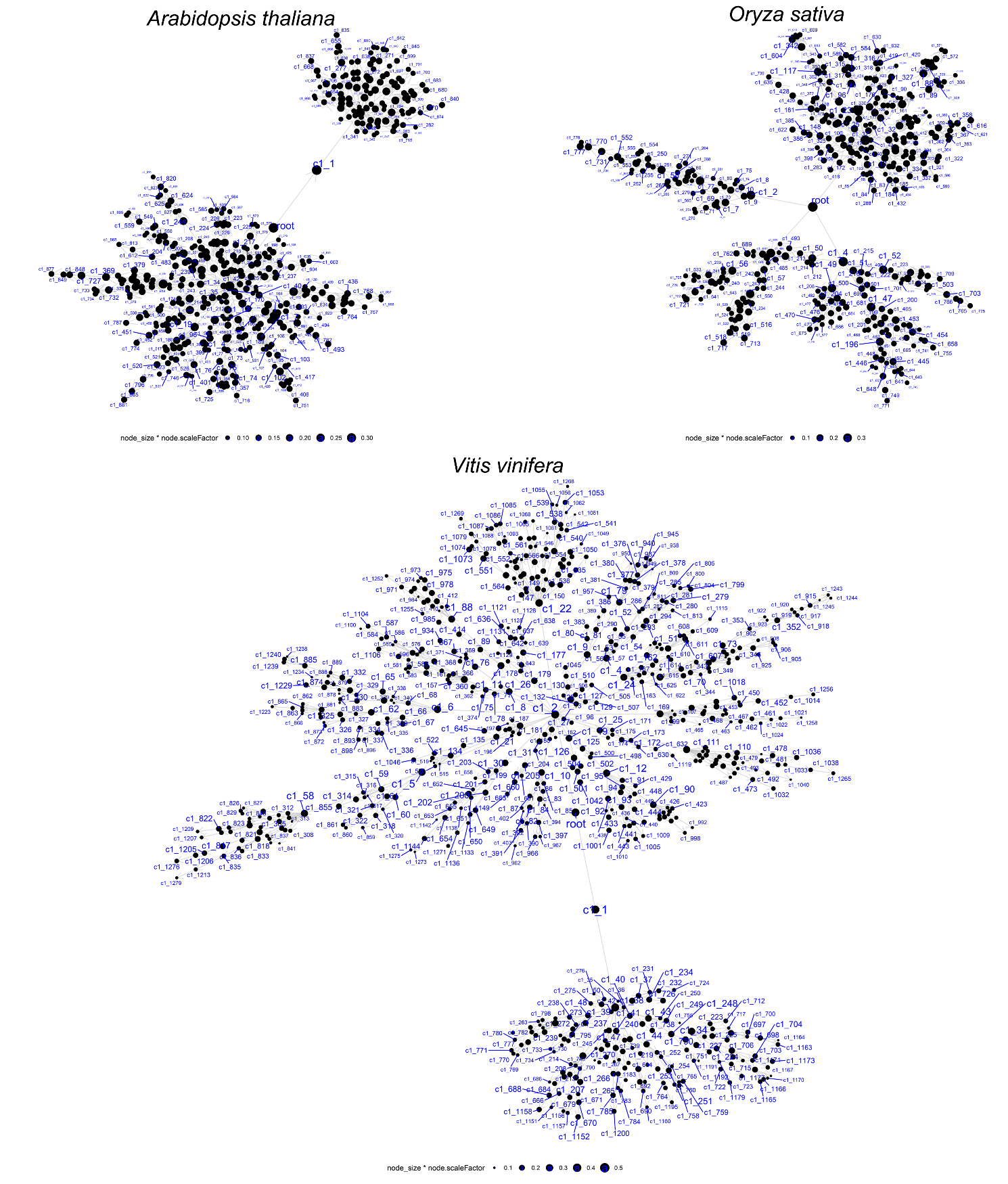


**Figure S1. Characterization of co-expression network modules in drought response in *A. thaliana*, *O.sativa*, and *V.vinifera*.** The circular nesting diagram showing the hierarchical structure of drought response network containing 21,420 genes and 455 modules in *A. thaliana*; 30,984 genes and 412 modules in *O.sativa*; 44,988 genes and 732 modules in *V.vinifera*.

**
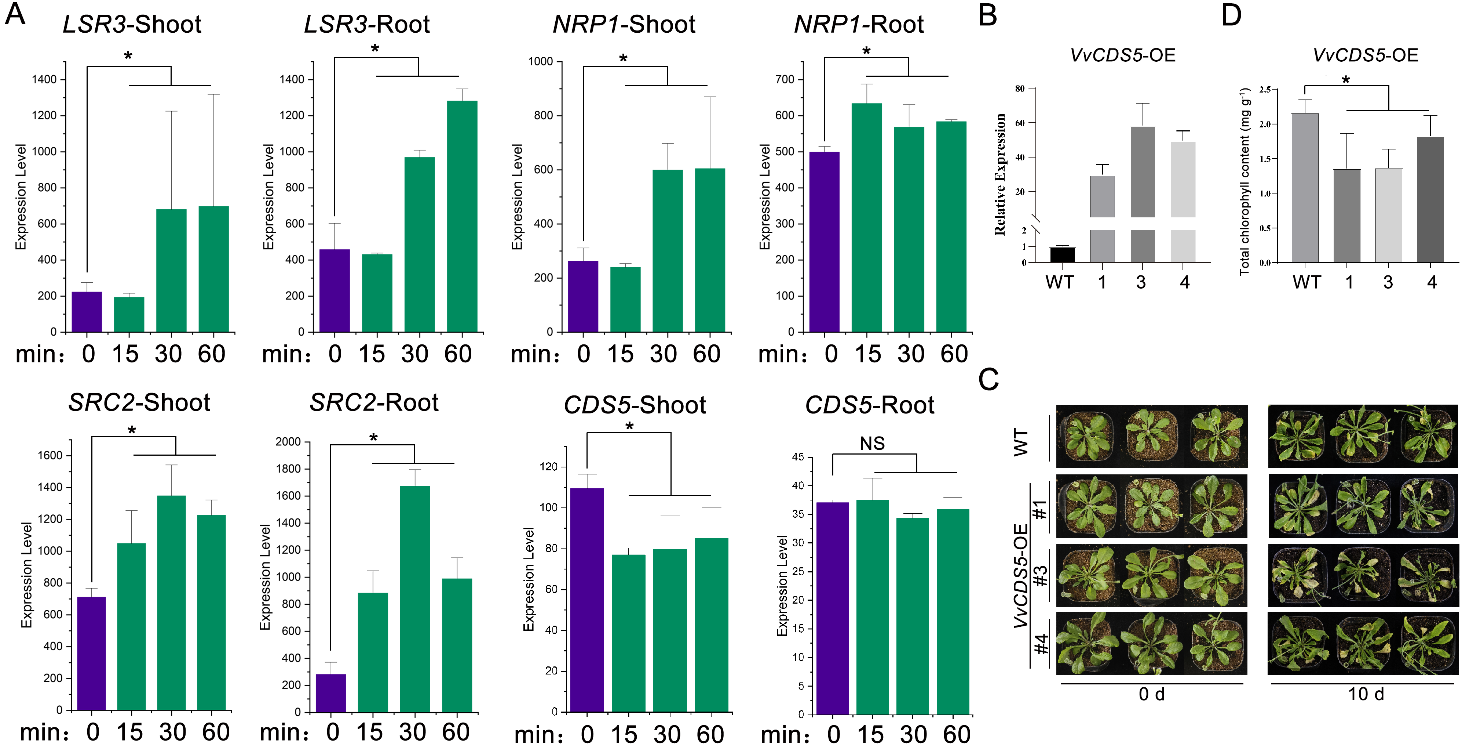
**

**Figure S2. Response of potential high-confidence drought-responsive genes to drought stress.** (A) Mean expression ± SEM (*n* = 3) of *LSR3*, *NRP1*, *SRC2,* and *CDS5* genes in the shoots and roots of plants after they experienced dehydration for a certain period of time (0, 15, 30, and 60 minutes). (B) q-PCR identification of VvCDS5 transgenic Arabidopsis, showing the relative expression levels of VvCDS5 in overexpression lines (*n* = 3). (C) Phenotypic analysis of Arabidopsis wild type (WT) and VvCDS5 overexpression lines (#1, #3, #4) under drought stress, with phenotypes recorded at 0 days, 10 days of drought. (D) Total chlorophyll content in shoot of Arabidopsis wild type (WT) and VvCDS5 overexpression lines (#1, #3, #4) under drought stress, with phenotypes recorded at 10 days of drought (*n* = 6). Asterisks indicate significant differences (*p* < 0.05; NS, not significant, one-way ANOVA followed by Duncan's multiple range test). Error bars represent standard errors.


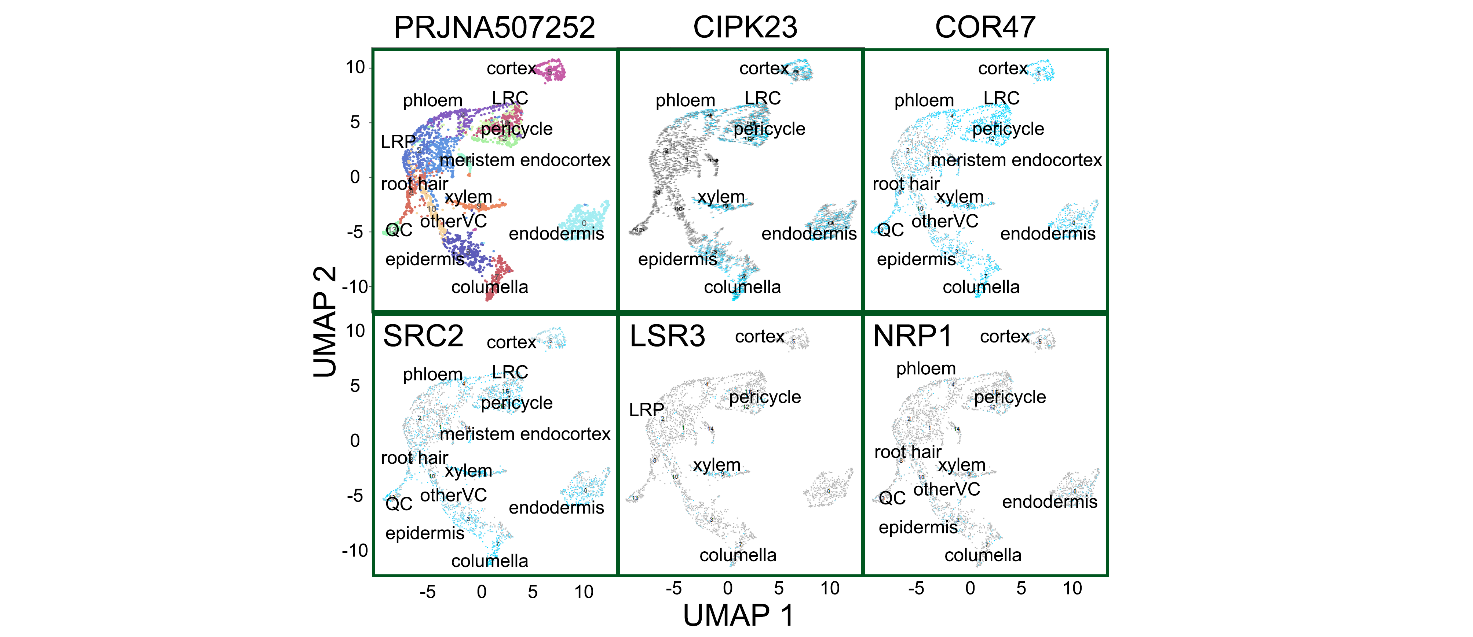


**Figure S3. Characterization of cell types of drought response genes.** UMAP plot of single cell transcriptome profiles (PRJNA507252). Various cell types were differentially colored. UMAP plot of single cell transcriptome profiles of *CIPK23*, *COR47*, *SRC2*, *LSR3*, and *NRP1*. LRC: lateral root cap; LRP: Lateral root primordium; QC: quiescent center; otherVC: other vascular cell.

**
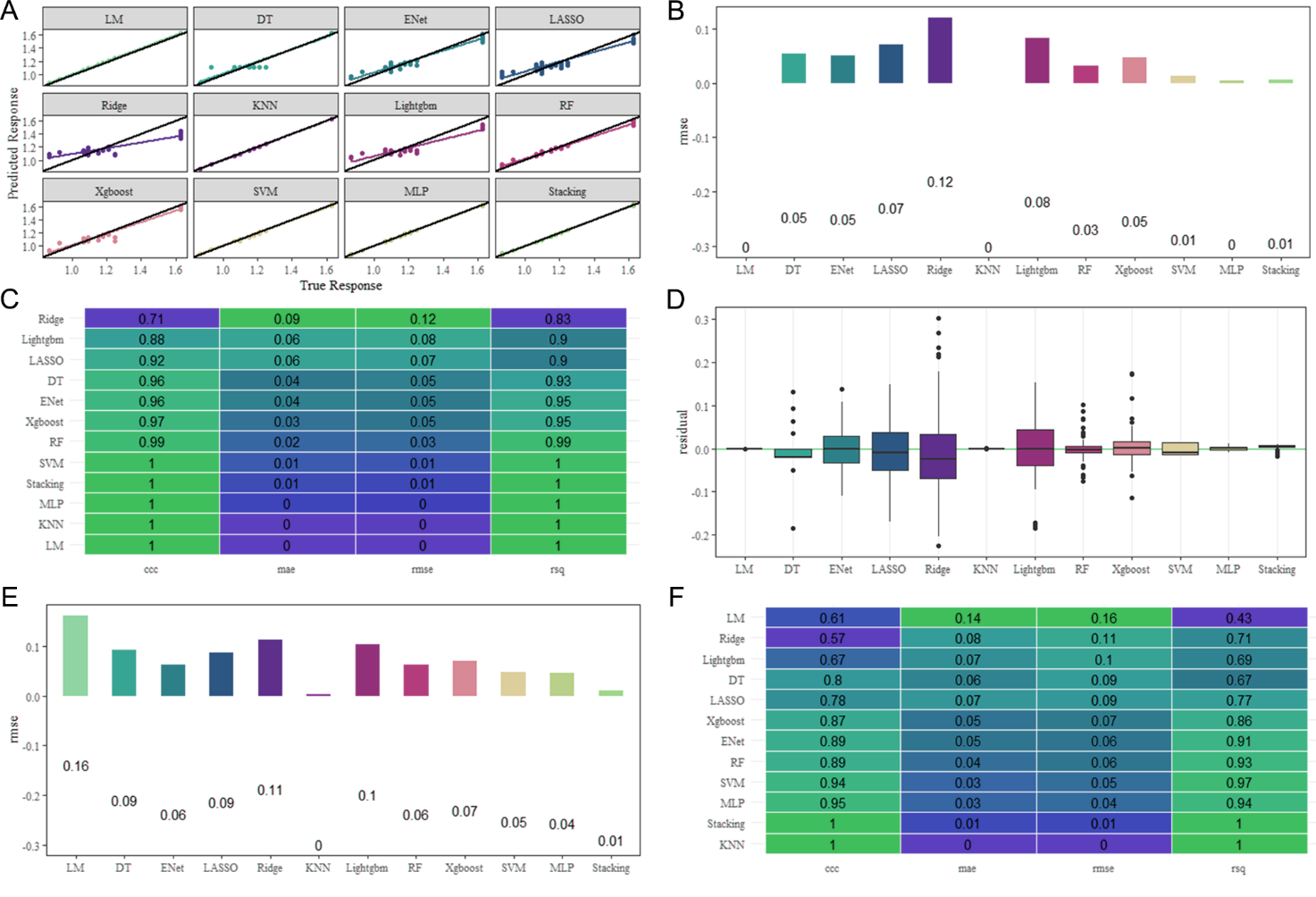
**

**Figure S4. Drought response key factor model based on machine learning.** (A) The scatter plot showing the relationship between the predicted values ​​and the true values ​​of the eleven models (LM, DT, ENet, LASSO, Ridge, KNN, Lightgbm, RF, Xgboost, SVM, MLP) on the training set. (B) The bar chart showing the residual distribution of the 11 models on the training set to evaluate the prediction error of the models. (C) The heat map showing Concordance Correlation Coefficient (CCC), Mean Absolute Error (MAE), Root Mean Square Error (RMSE), and R Squared (RSQ) of the 11 models on the training set. (D) The box plots showing the residual distributions of the eleven models to assess the prediction errors of the models on the training set. (E) The bar chart showing the residual distribution of the 11 models on the test set to evaluate the prediction error of the models. (F) The heat map showing Concordance Correlation Coefficient (CCC), Mean Absolute Error (MAE), Root Mean Square Error (RMSE), and R Squared (RSQ) of the 11 models on the test set.


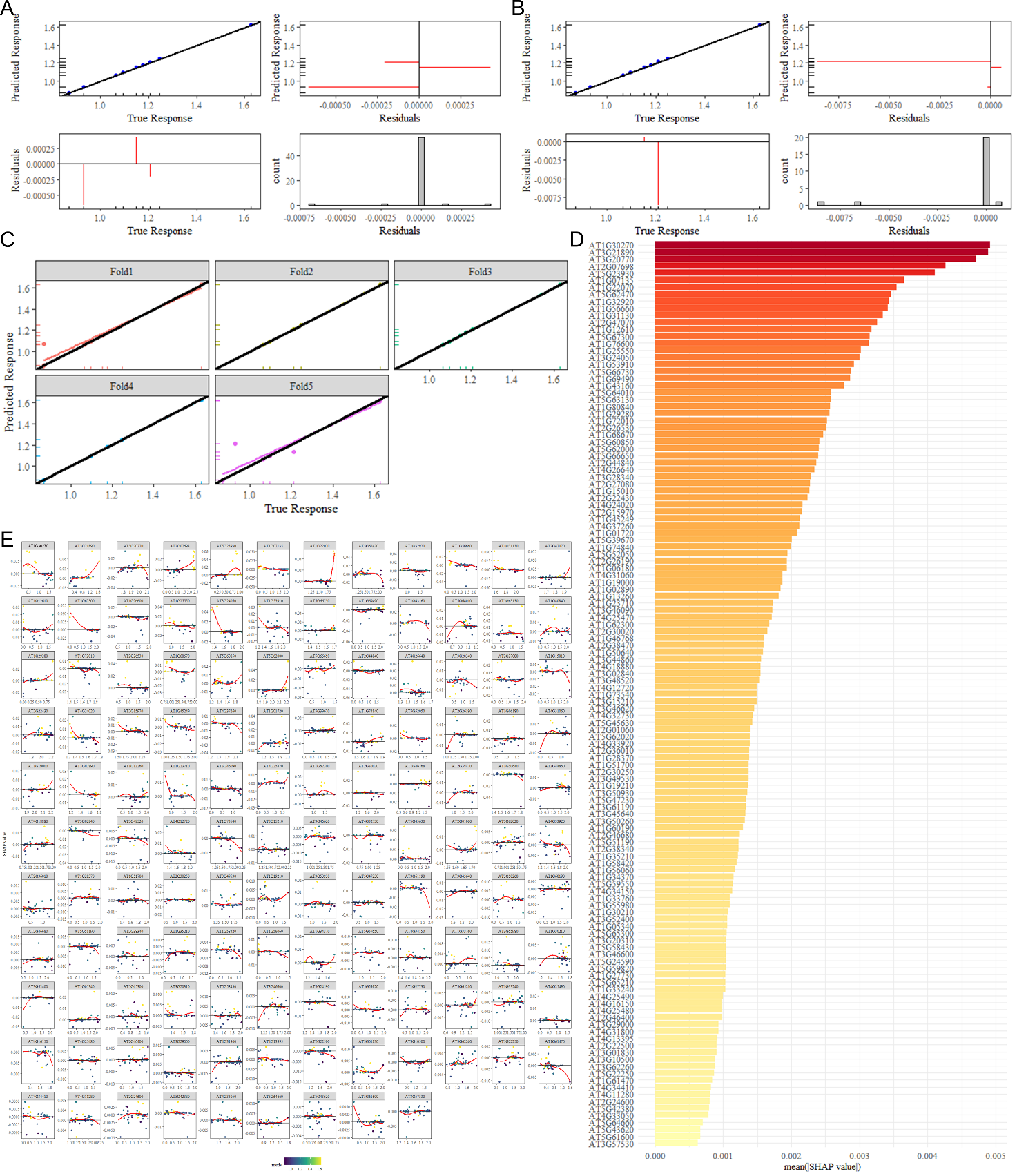


**Figure S5. Drought response key factor under the best-performing KNN model.** (A) showing the prediction error of the KNN model on the training set. (B) showing the prediction error of the KNN model on the test set. (C) The scatter plot showing the relationship between the predicted values ​​and the true values ​​of the KNN model. (D-E) showing the importance ranking of drought response factors under the KNN model based on SHAP values.


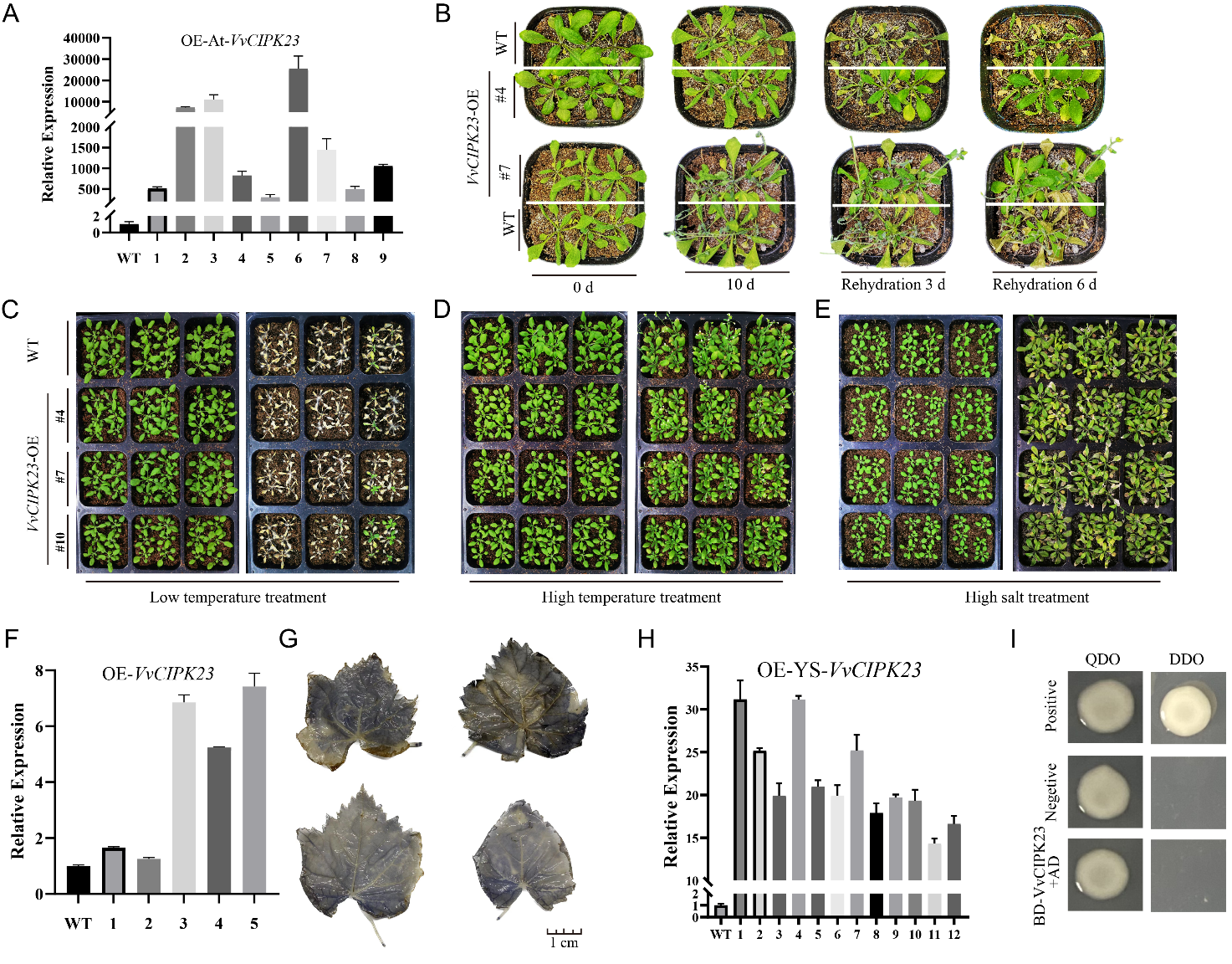


**Figure S6. Drought response key factor under the best-performing KNN model.** (A) q-PCR identification of VvCIPK23 transgenic Arabidopsis, showing the relative expression levels of VvCIPK23 in overexpression lines (*n* = 3). (B) Phenotypic analysis of Arabidopsis wild type (WT) and VvCIPK23 overexpression lines (#4, #7) under drought stress, with phenotypes recorded at 0 days, 10 days of drought, and 3 days, 6 days after rehydration. (C-E) Stress tolerance analysis of VvCIPK23 overexpression Arabidopsis lines (#4, #7, #10) under low temperature, high temperature, and high salinity conditions. (F) q-PCR analysis of relative expression levels of VvCIPK23 in transgenic grapevine leaves under different treatments (*n* = 3). (G) NBT staining of leaves from grapevine plantlets transiently expressing VvCIPK23 after drought stress, indicating oxidative damage in the leaves. (H) q-PCR identification of VvCIPK23 in transgenic grapevine callus, showing relative expression levels of VvCIPK23 (*n* = 3). (I) Yeast self-activation assay of VvCIPK23. pGBKT7-VvCIPK23 and pGADT7 were co-transformed into the yeast strain Y2H and cultured on DDO and QDO selective media to observe whether VvCIPK23 caused self-activation in yeast. Co-transformation of pGADT7-T and pGBKT7-p53 served as a positive control, while co-transformation of pGADT7-T and pGBKT7-Lam was used as a negative control. Error bars represent standard errors.


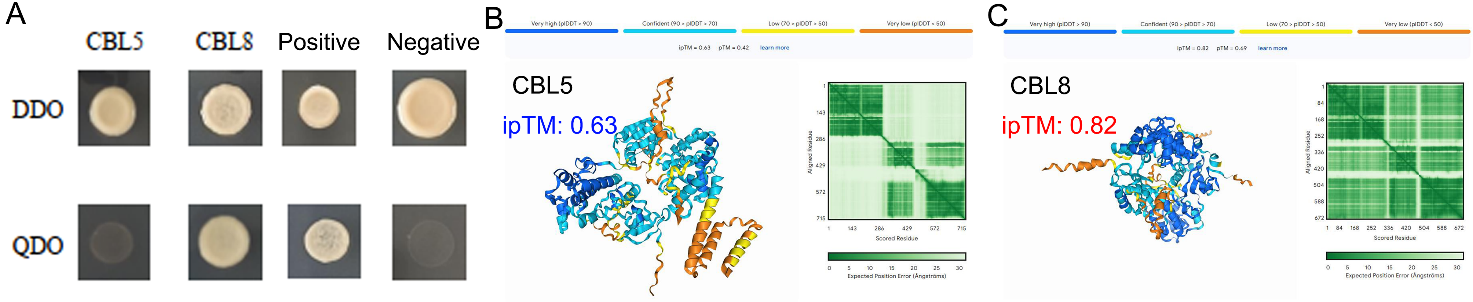


**Figure S7. Prediction and validation of protein-protein interaction between CIPK23 and CBLs.** (A) Yeast two-hybrid (Y2H) assay confirming the interaction between VvCIPK23 and VvCBL5/8. The PGBKT7-VvCIPK23 and PGADT7- VvCBL5/8 vectors were constructed, then co-transformed into the yeast strain Y2H after plasmid extraction and validate protein interactions on defective medium SD/-Trp/-Leu/-Ade/-His (QDO). The co-transformation of pGADT7-T and pGBKT7-p53 served as a positive control, while the co-transformation of pGADT7-T and pGBKT7-Lam was used as a negative control. (B-C) Verify the interaction of VvCIPK23 with VvCBL5/8 based on AlphaFold 3. The red box circles the part where the two proteins are hydrogen bonded and zooms in on the gray box. The interface predicted template modeling (ipTM) score are both derived from a measure called the template modeling score[41]. ipTM measures the accuracy of the predicted relative positions of the subunits within the complex. Values higher than 0.8 represent confident high-quality predictions, while values below 0.6 suggest likely a failed prediction. ipTM values between 0.6 and 0.8 are a gray zone where predictions could be correct or incorrect.
